# Navigating the Diagnostic Challenges of Posterior Circulation Ischaemic Strokes: A Case Report of Delayed Diagnosis

**DOI:** 10.7759/cureus.78403

**Published:** 2025-02-03

**Authors:** Hla Hla Aye, Eluzai Hakim

**Affiliations:** 1 Department of Acute Medicine, University Hospitals Dorset NHS Foundation Trust, Bournemouth, GBR; 2 Department of Stroke Medicine, University Hospitals Dorset NHS Foundation Trust, Bournemouth, GBR

**Keywords:** diagnosis challenge, migraine with aura, non-specific stroke presentation, posterior circulation ischaemic stroke, stroke misdiagnosis, vertigo

## Abstract

Posterior circulation infarcts (POCI) can present with non-specific symptoms, making diagnosis challenging and often delayed. We present a 58-year-old lady with a history of migraine who attended the emergency department of a district general hospital with sudden onset of vertigo, nausea, and vomiting but without focal neurological deficits. At the initial assessment, she experienced marked dizziness, which was worse on opening her eyes. Unenhanced brain computed tomography (CT) scan as well as CT angiography were normal. A provisional diagnosis of atypical presentation of migraine or benign paroxysmal positional vertigo was made. A stroke physician's opinion was sought, and in view of her persistent symptoms, magnetic resonance imaging (MRI) brain was carried out. This demonstrated three small acute infarcts: one in the posterior left temporal lobe, one in the inferior left occipital lobe, and one in the left cerebellar vermis. Further comprehensive stroke investigations were unremarkable. She was treated with dual antiplatelet therapy and statin. This case highlights the diagnostic challenges of posterior circulation ischaemic stroke and the importance of considering it in patients with unexplained vertigo, particularly those with stroke risk factors (migraine with aura, elevated cholesterol, and family history of stroke in this patient), to prevent misdiagnosis and serious complications.

## Introduction

Posterior circulation ischaemic stroke occurs when there is occlusion within the vertebrobasilar arterial system which supplies the brainstem, cerebellum, occipitoparietal lobes, and thalamus. It contributes to 20-25% of all ischaemic strokes [1-3]. The posterior circulation in the brain supplies an extensive area of brain tissue, and therefore, disruption can lead to a wide range of non-specific symptoms, making it challenging to locate posterior circulation strokes accurately [1]. Unlike anterior circulation strokes, which often present with more localized and recognizable symptoms, posterior circulation strokes involve structures responsible for complex and diffuse neurological functions, further contributing to diagnostic challenges [1]. Posterior circulation strokes are estimated to be three times more likely to be misdiagnosed than anterior circulation strokes in the hospital setting and is potentially life-threatening [1,2]. Early diagnosis and treatment of posterior circulation strokes are critical, as delayed or incorrect diagnosis can lead to serious complications, including severe disability or death [3].

Commonly used stroke screening tools, such as the Face Arm Speech Time (FAST) criteria, are less effective in identifying posterior circulation strokes because these strokes often lack the hallmark symptoms associated with anterior circulation strokes. Below, we present and discuss a patient of posterior circulation ischaemic stroke presenting with atypical symptoms leading to an initial incorrect diagnosis.

## Case presentation

A 58-year-old lady, with a past medical history of osteopenia and migraine with visual aura, presented to the emergency department (ED) of a district general hospital two hours after a sudden onset of vertigo while she was out for a run. The vertigo was associated with nausea and vomiting and worsened by eye-opening. She denied a history of preceding or current headache, limb weakness, speech disturbance, facial droop, preceding neck pain, or other neurological symptoms, apart from feeling unsteady on her feet. She had been investigated for palpitations over the past few years under the care of a cardiologist. She had multiple cardiac monitorings, which only revealed brief episodes of sinus tachycardia, all of which were asymptomatic. Her electrophysiology studies were negative for arrhythmias, and no further cardiac interventions were suggested. 

She takes sumatriptan for migraine on an as-required basis, but she had not taken any prior to this event. Her grandfather and paternal aunt suffered from strokes in their 50s, and her cousin in her 60s. She denied a history of miscarriages and has two healthy children. She drinks small amounts of alcohol on social occasions, is a non-smoker of tobacco products, and does not use recreational drugs.

**Figure 1:**
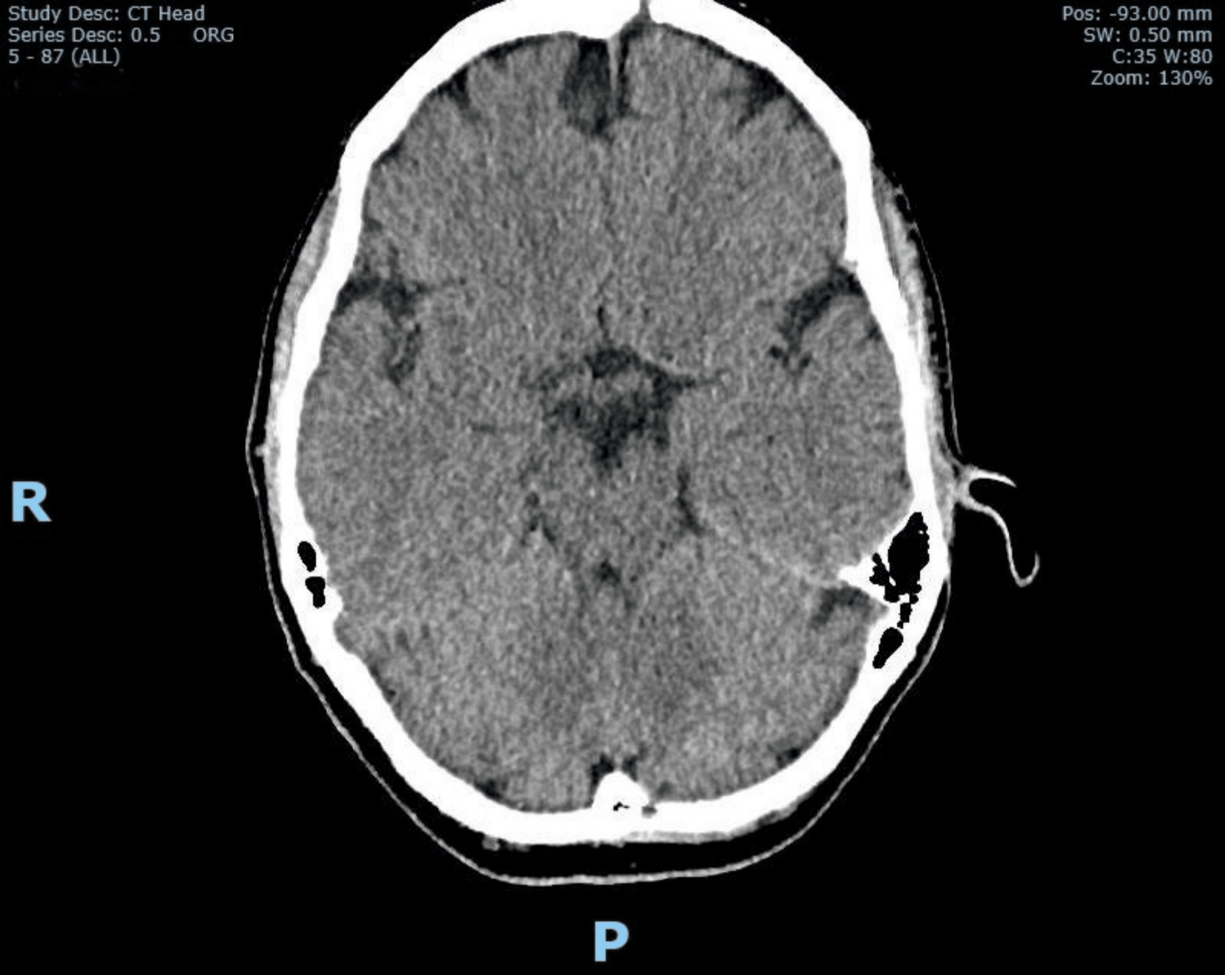
Normal unenhanced CT brain CT: computed tomography

Clinical examination revealed an alert patient who kept her eyes closed due to nausea. Power in all limbs and cranial nerves examination were normal. There was no incoordination in the finger-nose testing and heel-shin testing. There was no nystagmus, sensory deficits, or visual field defects, though she struggled to keep her eyes open during visual field assessments due to nausea. She was too unwell to have her gait assessed because of nausea and severe vertigo. The National Institute of Health Stroke Scale (NIHSS) was 0 (where 0 denotes no deficits and 42 indicates severe disability). Examination of the cardiac, respiratory, and abdominal systems was unremarkable. The non-contrast CT (NCCT) of the brain obtained at the emergency department was normal (Figure 1). A CT angiogram from the aortic arch to the circle of Willis showed no identifiable major vessel occlusion or evidence of vertebral and basilar arterial dissection or occlusion. There was also no evidence of significant atherosclerosis shown in the carotids and intracranial vessels. The initial working diagnosis was atypical presentation of migraine or benign paroxysmal positional vertigo (BPPV), for which she was treated with prochlorperazine to control her symptoms of vertigo and nausea, and an opinion was sought from the duty stroke team.

**Figure 2:**
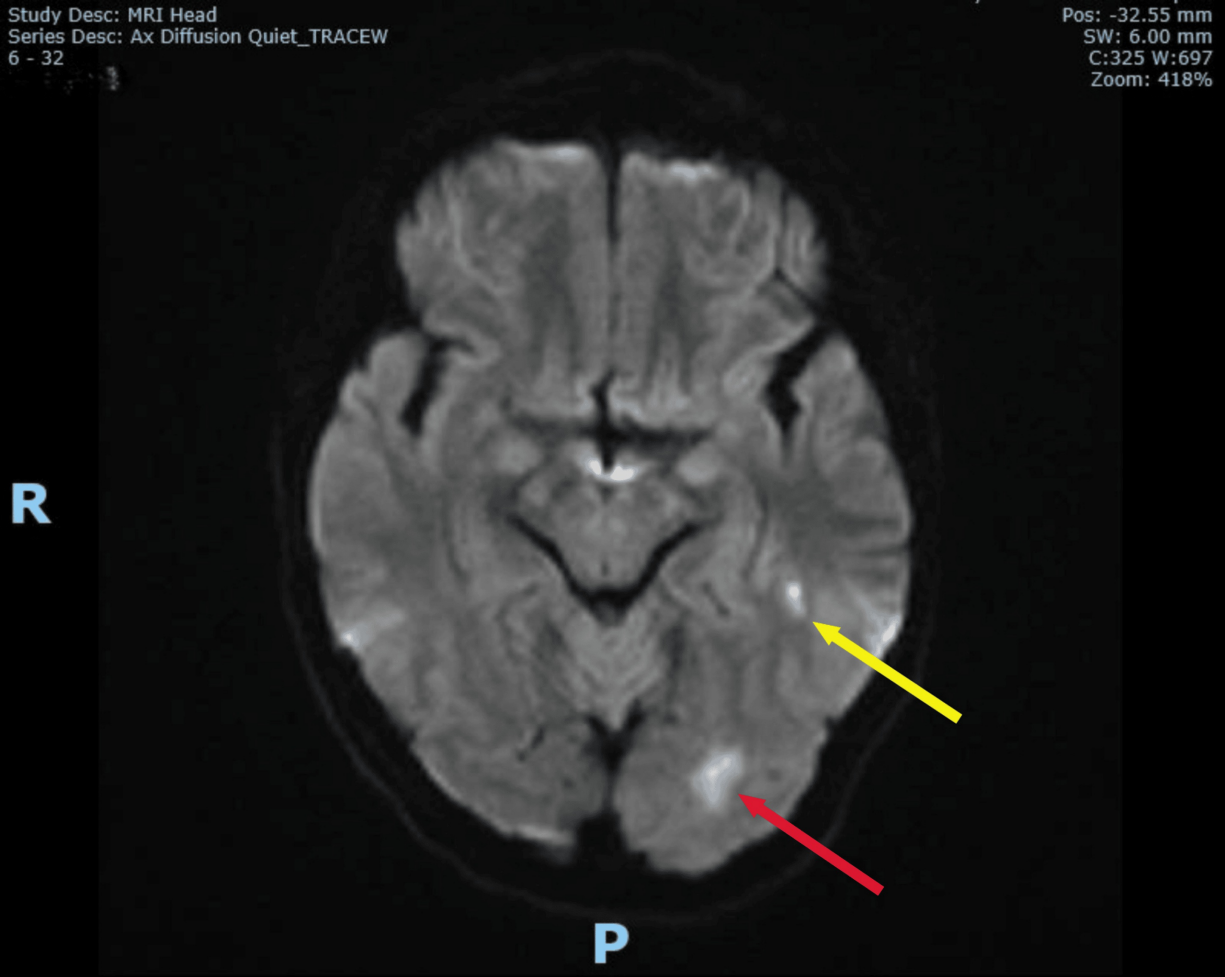
MRI brain DWI showing infarcts at posterior left temporal lobe (yellow arrow) and inferior left occipital lobe (red arrow) MRI: magnetic resonance imaging DWI: diffusion-weighted imaging

**Figure 3:**
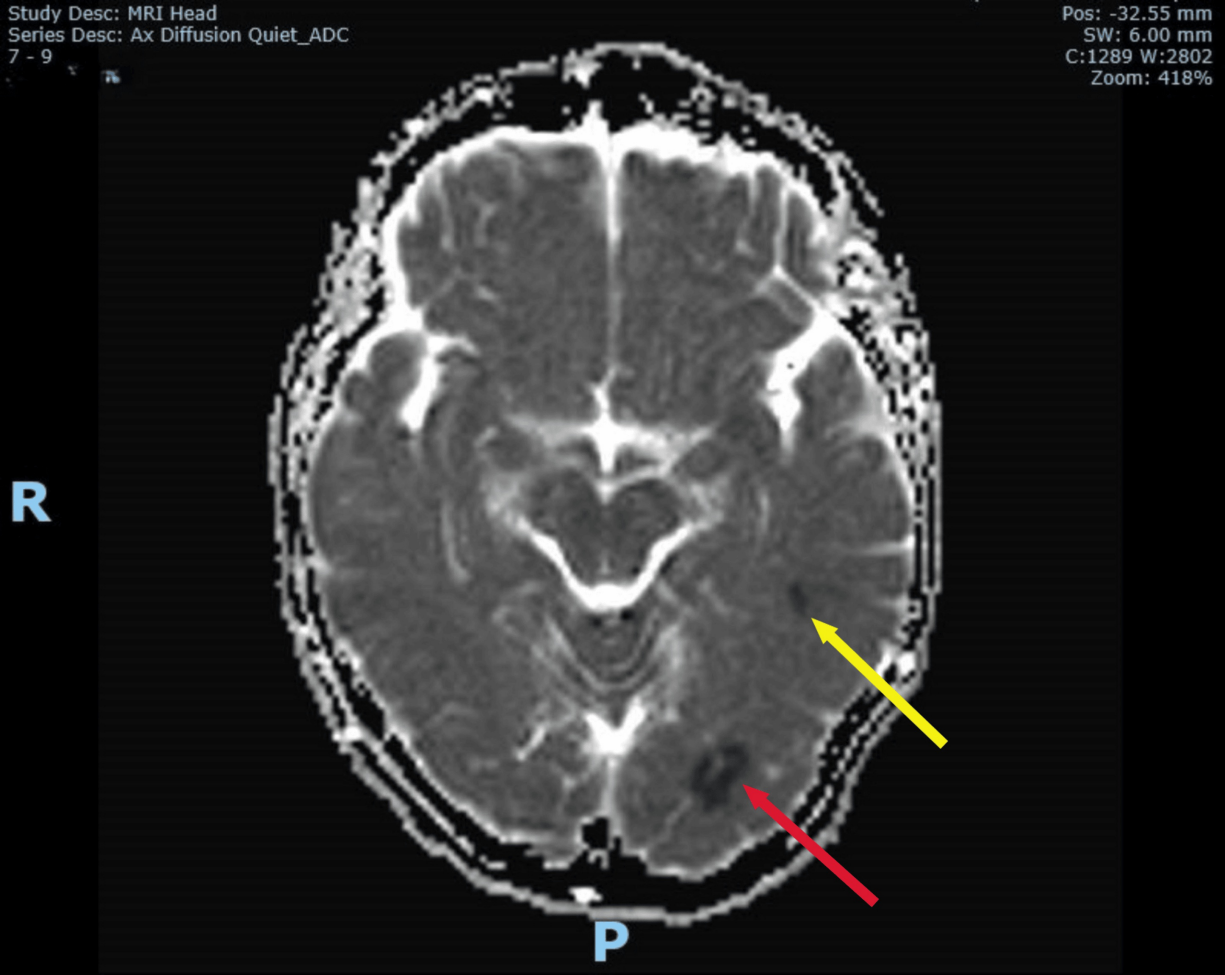
Corresponding lesions shown on MRI brain ADC confirming acute left posterior temporal (yellow arrow) and inferior occipital (red arrow) infarcts MRI: magnetic resonance imaging ADC: apparent diffusion coefficient

**Figure 4:**
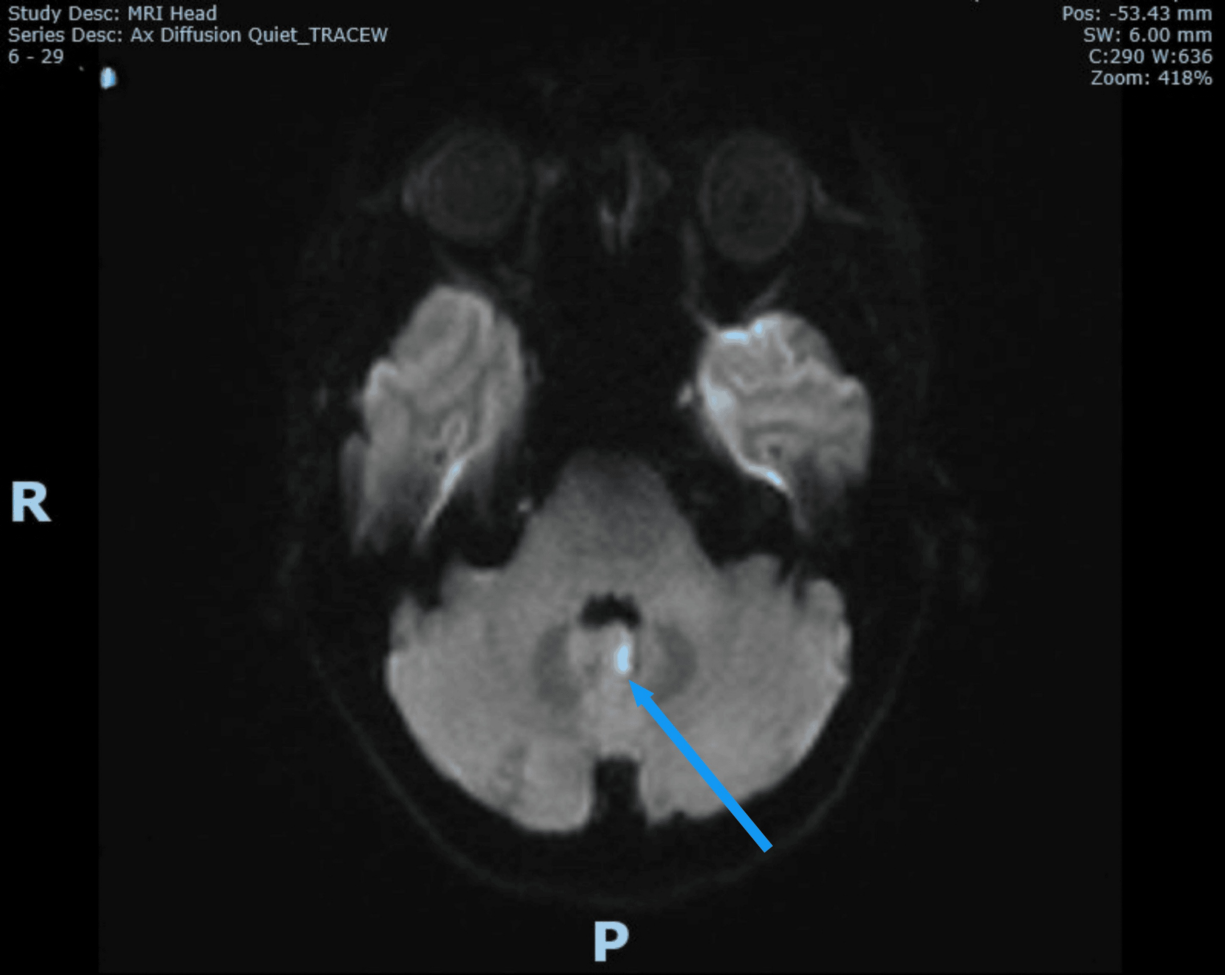
MRI brain DWI showing infarct at left cerebellar vermix (blue arrow) MRI: magnetic resonance imaging DWI: diffusion-weighted imaging

**Figure 5:**
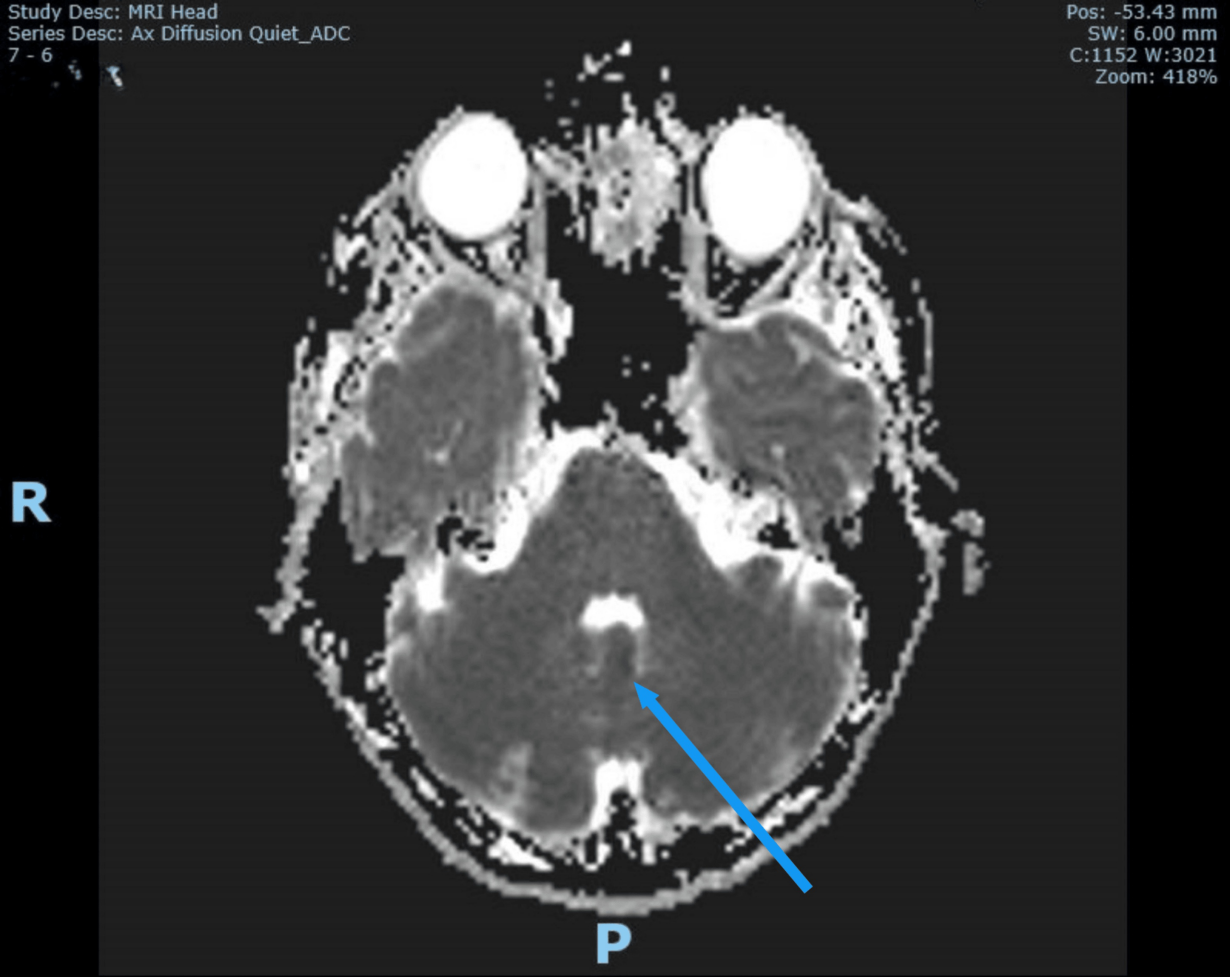
Corresponding MRI brain ADC confirming acute left cerebellar vermix infarct (blue arrow) MRI: magnetic resonance imaging ADC: apparent diffusion coefficient

Upon review of the patient by the duty stroke physician four hours after admission (six hours after onset), no signs suggestive of posterior circulation stroke, such as cranial nerve deficits, cerebellar signs, brainstem dysfunction, or visual field defects, were identified. Gait could not be assessed due to her vertigo and nausea. However, due to her ongoing severe vertigo and inability to keep her eyes open for a prolonged period during the assessment five hours after admission, a magnetic resonance imaging (MRI) brain was arranged and completed six hours after admission (eight to nine hours after onset). The MRI revealed three small acute infarcts: one in the posterior left temporal lobe, one in the inferior left occipital lobe, and one in the left cerebellar vermis (Figures 2-5). This confirmed the diagnosis of acute multiple posterior circulation infarctions (POCI).

Her blood tests were unremarkable. Serum cholesterol was 4.8 mmol/l (normal level for our laboratory is <5.2 mmol/L), and low-density lipoprotein (LDL) cholesterol was 2.6 mmol/l (normal range). Other investigations included a 12-lead electrocardiogram (ECG), an echocardiogram looking for a proximal source of emboli, 24-hour telemetry, and a young stroke screen to look for predisposing factors for stroke and includes the following: erythrocyte sedimentation rate (ESR), lupus anticoagulant, connective tissue disease screen, myeloperoxidase (MPO) and proteinase 3 (PR3), antineutrophil cytoplasmic antibodies (ANCA), alpha-galactosidase, complement C3 and C4, and homocysteine. These were all within the reference range for our laboratory.

She was admitted to the stroke unit and started on dual antiplatelet therapy with aspirin 75 mg orally once daily for 21 days, concurrently with clopidogrel 75 mg orally once daily long term. Atorvastatin 40 mg orally once daily was added to the treatment. She was assessed by the therapy team and offered physical as well as occupational therapy. Her symptoms gradually improved over a three-day period, and she was discharged home with plans to offer her therapy at home through the Early Supported Discharge (ESD) scheme that our unit operates. She will be followed up in our post-discharge stroke review clinic in three months.

## Discussion

The prompt diagnosis of posterior circulation strokes at presentation continues to be challenging for clinicians due to the varied anatomical structures involved and the complexity of the vertebrobasilar vasculature.

The anatomy of the posterior circulation includes the vertebral arteries, the basilar artery, three paired cerebellar arteries, and posterior cerebral arteries [1]. Unlike the internal carotid arteries, which give rise to numerous smaller branches, the bilateral vertebral arteries combine to form a single basilar artery that supplies the brainstem, occipital lobes, and thalamus [1]. In contrast to the anterior circulation, the posterior circulation relies more on penetrating vessels, which lack collaterals. Various vascular pathologies can lead to multi-level strokes in distinct anatomical regions of the posterior circulation. Due to the complexity of the brainstem and its arterial distribution, accurately localising clinical signs and determining infarct sites in posterior circulation strokes can be challenging [1].

Our case illustrates these diagnostic difficulties, where the patient’s vertigo, nausea, and vomiting initially suggested a benign aetiology rather than a stroke, delaying an accurate diagnosis.

According to a 2012 analysis by the New England Medical Centre Posterior Circulation Registry, the most common presenting symptoms of posterior circulation strokes are dizziness (47%), unilateral limb weakness (41%), dysarthria (31%), headache (28%), nausea or vomiting (27%), and blurry vision (20%). The most common clinical signs are unilateral limb weakness (38%), gait ataxia (31%), unilateral limb ataxia (30%), dysarthria (28%), nystagmus (24%), and a positive Babinski’s sign (24%) [3-5].

The FAST test, commonly used as a stroke screening tool in the community, is less effective at detecting posterior circulation strokes compared to anterior circulation strokes [2,3]. The modification of the FAST test by adding "balance" and "eyes," which highlight sudden gait disturbances and visual symptoms, resulting in the BEFAST acronym, has been found to decrease the rate of missed strokes when compared to the traditional FAST test [6]. The “Five Ds”, which focus on symptoms such as Dizziness, Diplopia, Dysarthria, Dysphagia, and Dystaxia, have been used to describe the signs and symptoms of POCI. This mnemonic was introduced based on findings from the New England Medical Center Posterior Circulation Registry, which provides a comprehensive analysis of the symptoms and signs associated with posterior circulation ischemia, forming the basis for the "5 Ds" mnemonic [5].

The HINTS test (Head Impulse, Nystagmus, Test of Skew) has proven reliable for distinguishing posterior circulation strokes from peripheral vestibular disorders in patients presenting with acute vestibular syndrome (AVS). According to a prospective, cross-sectional study, HINTS is more sensitive than early MRI in detecting stroke in patients with acute vestibular syndrome [7]. The NIHSS, which is primarily designed to focus on anterior circulation stroke signs and symptoms, may underestimate the severity of posterior circulation strokes [1,4,8]. A study comparing the expanded NIHSS (e-NIHSS) with the original NIHSS in posterior circulation strokes shows that the e-NIHSS is more effective for diagnosis and prognosis and recommends its inclusion in future clinical guidelines [9].

Conditions that can mimic POCI include acute peripheral vestibular dysfunction, intracerebral haemorrhage, brain tumour, basilar migraine, toxic or metabolic disturbances, posterior reversible encephalopathy syndrome, neuroinflammatory or chronic infectious disorders, and infection of the medulla, pons, and cerebellum [3,4]. Prompt MRI imaging is essential to differentiate these conditions from cerebrovascular events.

An unenhanced CT head is typically the first step in evaluating suspected posterior circulation stroke. While CT is very sensitive to detecting haemorrhage, it may miss acute ischaemic strokes. MRI is more sensitive for detecting POCI, making it valuable when CT results are negative but symptoms suggestive of POCI persist. Vascular imaging, such as CT or MR angiography, is essential for assessing potential occlusions or dissections and guiding further treatment decisions [4].

Treatment

Reperfusion therapy using either intravenous thrombolysis (IVT) alone or in combination with endovascular thrombectomy (EVT) is essential for treating anterior and posterior circulation strokes. Although recent trials demonstrate the advantages of EVT in improving outcomes for POCI secondary to basilar artery occlusion, optimal patient selection and the use of bridging IVT prior to EVT require further studies to establish evidence-based guidelines [8]. Aspirin 300 mg is recommended for patients with acute ischaemic stroke within 24-48 hours of symptom onset, and clopidogrel is an alternative anti-platelet agent for those with allergies to aspirin [8]. In patients with a minor ischemic stroke or high-risk transient ischemic attack, dual antiplatelet therapy with clopidogrel and aspirin may reduce the likelihood of recurrent strokes during the initial three months [10].

Neurosurgical intervention (including external ventricular drainage or decompression) may be lifesaving in large-volume cerebellar infarction with raised intracranial pressure or acute hydrocephalus [3,8].

Secondary prevention

Secondary prevention after POCI includes antiplatelets, anticoagulants for patients with atrial fibrillation, statins as an adjunct to the above medications, judicious blood pressure management, and modification of risk factors [3,8]. Vertebral artery stenting is controversial, with limited evidence supporting its use in stenosis or dissection [8].

## Conclusions

This case underscores the challenges in diagnosing posterior circulation strokes, which often present with non-specific symptoms like vertigo, nausea, and vomiting. The initial misdiagnosis of benign conditions, such as atypical migraine or benign paroxysmal positional vertigo, can cause delayed treatment, potentially resulting in severe complications. Clinicians should maintain a high index of suspicion for POCI in patients with unexplained vertigo, especially those with stroke risk factors. Recognising the limitations of standard stroke screening tools like the FAST test and NIHSS is important.

Early MRI is crucial in detecting posterior circulation infarcts when the initial CT results are inconclusive. Prompt recognition and management of POCI can prevent severe complications and improve patient outcomes.
